# Dimensions of the COVID-19 pandemic: prevalence of common mental disorders in "invisible" health workers and their association with occupational stressors

**DOI:** 10.1590/1980-549720240039

**Published:** 2024-07-29

**Authors:** Manuela Matos Maturino, Camila Carvalho de Sousa, Lusicleide Galindo da Silva Moraes, Danyella Santana Souza, Maria Yaná Guimarães Silva Freitas, Tânia Maria de Araújo

**Affiliations:** I Universidade Estadual de Feira de Santana, Epidemiology Center – Feira de Santana (BA), Brazil.; II Bahia State Secretariat of Health, Eastern Regional Health Center – Salvador (BA), Brazil.; III Hospital Universitário Professor Edgar Santos – Salvador (BA), Brazil.; IV Universidade Estadual de Feira de Santana, Integrated Public Health Research Center, Health Surveillance Research and Extension Center – Feira de Santana, Bahia, Brazil.

**Keywords:** COVID-19, Healthcare worker, Occupational stress, Mental health

## Abstract

**Objective:**

To evaluate the association between occupational stressors and common mental disorders (CMD) among "invisible" health workers in the context of the COVID-19 pandemic.

**Method:**

Cross-sectional study including a probabilistic sample of 1,014 health workers from three municipalities in Bahia. CMDs were assessed using the SRQ-20. The Effort-Reward Imbalance (ERI) scale and the Demand-Control Model assessed occupational stressors. Descriptive, bivariate, and multiple analysis to evaluate the association between the variables of interest.

**Results:**

The global prevalence of CMD was 39.9%; it was higher among CHA/EDCA (47.2%), followed by management and surveillance staff (38.6%), technicians (35.4%), and support/maintenance/cleaning staff (29.9%). The association between occupational stressors and CMD varied among occupations: 1. Excessive work commitment (EWC), effort-reward imbalance (ERI), and psychological demand were associated with CMD among support/maintenance/cleaning workers; 2. EWC and ERI were associated with CMD among CHA/EDCA; 3. EWC, ERI, and low control over work were associated with CMD among technicians; 4. Among management and surveillance workers, only ERI remained associated with CMD.

**Conclusions:**

Occupational stressors played a relevant role in mental illness, with variation between occupational strata, demanding attention, monitoring, and control.

## INTRODUCTION

During health crises, healthcare workers (HW) often feel helpless and overwhelmed by their responsibilities, experiencing physical and psychological strain. Intensification of the pace and demand of work; drastic changes in work routine; absence or limitation of support and unfavorable working conditions, with an increased level of exposure to the risk of becoming ill and dying, were situations highlighted by the emergence of the new disease, COVID-19^1^.

Healthcare services, essential for combating and managing a pandemic, were among the few that remained operational in person throughout the period. In Brazil, the Unified Health System (*Sistema Único de Saúde* – SUS) is primarily responsible for implementing measures and providing health services to mitigate the impact of COVID-19. During a health crisis like a pandemic, the lives of entire populations depend on the organization and actions undertaken by these workers^2,3^.

The mental health of HW has been the focus of several studies in recent years, with particular attention to common mental disorders (CMD). These disorders are characterized by symptoms such as insomnia, anxiety, fatigue, irritability, depressive mood, difficulty concentrating, and somatic complaints^4^. Although they do not constitute a specific diagnostic category, they meet the criteria for disorders listed in the International Classification of Diseases (ICD-10) and the Diagnostic and Statistical Manual of Mental Disorders (DSM)^5^.

High psychosocial demand is one of the most frequently identified factors negatively impacting the mental health of HWs, particularly due to occupational stressors^1,6^. This psychosocial dimension arises from the nature of their work, including direct contact with difficult patients, long working hours, fear of making errors during care, and precarious working conditions, all of which are experienced as sources of stress, leading to overload and chronic stress^6,7^. These factors were exacerbated by the pandemic, making HW more vulnerable to psychological illness.

Despite the high occupational exposure and crucial role of various occupational categories in the health sector, a significant portion of these workers remain invisible, underrecognized, and overlooked in studies evaluating health problems among HWs. These individuals are referred to as "invisible workers." Though they perform essential activities, their functions are often undervalued and forgotten by society, and they frequently go "unnoticed" by their own teams, institutions, and society at large^8,9^.

This study focuses on the analysis of these "invisible" health workers, categorized into four groups based on occupational demands:

1. Support, maintenance, and cleaning workers (ambulance drivers, maintenance staff, operational support, cleaning staff, kitchen staff, and administration);
2. Technicians (nursing, oral health, radiology, laboratory, and clinical analysis technicians and assistants);
3. Community Health Agents (CHA) and Endemic Diseases Control Agents (EDCA); and
4. Health management and surveillance (sanitarians, health inspectors, surveillance technicians, institutional supporters, managers, and coordinators).

This study examines the mental health of HWs and explores the relationship between the psychosocial characteristics of work and mental illness observed during the pandemic. The aim of the study was to assess the association between occupational stressors and the occurrence of common mental disorders among "invisible" health workers within the context of the COVID-19 pandemic.

## METHODS

The study is a cross-sectional investigation conducted in three municipalities located in the interior of Bahia, associated with the research project "Surveillance and Monitoring of Infectious Diseases in the Health Sector," which received financial support from CNPq under grant number 427045/2016-9. The project was approved by the Ethics Committee for Research with Human Beings under protocol CAAE 90204318.2.0000.0053.

Sample defined in successive stage procedures: nominal list of active workers; sample size estimation; sample size definition; sample stratification; random selection of workers (SPSS, version 23.0).

The sample size was calculated using the total number of workers (4,849) across the three municipalities. With an incidence of work accidents among HW of 42%, a confidence level of 95%, and a precision of 3%, resulting in a minimum required sample of 857 workers. Additionally, 20% was added to this number to account for potential losses and refusals, resulting in a final sample size of 1,028 workers.

This study investigates the correlation between occupational stressors and mental disorders among "invisible" healthcare workers. Not included in this analysis are doctors, nurses, dentists, physiotherapists, psychologists, nutritionists, physical educators, occupational therapists, and pedagogues.

Data collection occurred between April/2021 and April/2022, involving face-to-face interviews conducted at workplaces. A questionnaire, developed based on a literature review, comprising 8 blocks, was utilized:

1. General identification;
2. General information about the job;
3. Environment/workplace conditions;
4. Psychosocial characteristics of work and mental health;
5. Household chores;
6. Lifestyle habits and health-related aspects;
7. Arboviruses; and
8. Violence.

Trained staff conducted data quality control and database entry. Biosafety measures were implemented during data collection to ensure safety amid the pandemic.

CMD, the outcome variable, was evaluated using the Self-Reporting Questionnaire (SRQ-20). To screen for CMD, a cutoff point of seven or more positive responses for women and five or more for men was utilized, following recommendations from a validation study of SRQ-20^10^.

The psychosocial aspects of work, specifically occupational stressors, were assessed using two models: the Effort-Reward Imbalance (ERI) model, developed by Siegrist^11^, and the Demand-Control Model (DCM), proposed by Karasek^12^ (measurement instruments tested on a Brazilian worker population, presenting good performance).

The ERI Model^11^ evaluates two main dimensions: effort (energy expended in carrying out work tasks, encompassing both physical and psychological exertion) and reward at work (including financial gains, perception of recognition and respect in the work environment, expectations of promotion, alignment of job position with training, and the sense of fairness in interpersonal relationships). Additionally, the model incorporates a third dimension (excessive commitment to work), reflecting an intrinsic, subjective factor characterized by striving for recognition and approval^13^. The ERI questionnaire consists of 23 items [effort scale: 6 items; reward: 11 items and excessive commitment to work (over-commitment): 6 items]. Participants respond to each item on a scale ranging from 1 (strongly disagree) to 4 (strongly agree). Scores for each scale are calculated by summing its respective items. The second tertile was used as a cutoff point to dichotomize the scales proposed for the composition of effort (low/high), reward (low/high), and excessive commitment (absent/present) (Chart 1). The effort-reward imbalance score is computed using the formula: ERI=Effort score x correction factor/reward score. Scores above 1 indicate an effort-reward imbalance.

DCM^12^ emphasizes two core elements in the workplace: psychological demand and control. Psychological demand pertains to the mental challenges encountered by workers during the performance of their duties (pace of work, adequacy of time allocated, workload volume, among others). Control refers to the extent of autonomy that workers possess in decision-making processes related to their work^14^. Combining these elements results in the identification of four distinct work situations: low demand (high control/low demand), active work (high demand/high control), passive work (low demand/low control), and high demand (high demand/low control).

The main hypothesis of this model is that highly demanding work presents the greatest psychosocial risk to physical and mental health^13^. Social support, later included in the DCM, is considered a third dimension. It is believed that assistance from colleagues and superiors in carrying out tasks, social integration, and trust within the group play significant roles in health outcomes.

The Job Content Questionnaire (JCQ)^16^ is the instrument designed to measure DCM scales. A validation study on formal and informal workers in Brazil identified good overall performance of the instrument for investigating psychosocial aspects of work^16^. The JCQ scales were dichotomized using the median as the cutoff point to define high and low categories (Chart 1), thereby establishing specific work situations: low demand, active work, passive work, and high demand^12,14^.

**Chart 1:** Dimensions of the demand-control model and effort-reward imbalance model, number of items, score variations, and cutoff points used.

Dimensions		No. of items	Variation	Cutoff point	
				Median	2^nd^ tertile
Demand-control model					
	Psychological demand	5	22–68	42.0	
	Control over work	9	32–92	62.0	
	Social support at work	6	6–28	18.0	
Effort-reward imbalance model					
	Effort	6	6–24		15
	Reward	11	19–42		30
	Excessive commitment	6	6–24		16

In addition to occupational stressors measured by JCQ and ERI, the study also considered socioeconomic factors (gender, age, education, race/skin color, marital status, children, income) and labor factors (work hours, employment relationship, occupation, length of time in the profession, and activities compatible with the position).

For the general characterization of the sample, descriptive analyses were conducted, taking into account the previously defined occupational groups of the "invisible" workers. The analysis of the association between occupational stressors and the occurrence of CMD was stratified by occupation. Prevalence ratios (PR), 95% confidence intervals (CI), and p-values were calculated using Pearson's χ^2^ test (Statistical Program for the Social Sciences 23.0/SPSS 15.0 and OpenEpi 3.0).

The final regression models were estimated separately for the stressor groups (effort-reward imbalance and demand-control model) and for the occupational groups analyzed. Poisson regression with robust variance was used to estimate prevalence ratios, 95% confidence intervals, and p-values in the multiple (multivariable) analysis^17,18^.

In selecting variables for multiple analysis, Pearson's chi-square test (χ^2^) was employed, considering all variables in a non-conditional manner. The significance level for entry into the multivariate model was set at 25%, using the likelihood ratio test. The backward method was used to select the variables, with a 5% significance criterion for variables to remain in the final model. The quality of fit for the final model was diagnosed using the Hosmer and Lemeshow test, ROC curve, and analysis of influential observations. Data Analysis and Statistical Software (STATA), version 12.0, was used for this stage.

## RESULTS

The sample consisted of 1,014 HW, of which 795 were in invisible occupations, representing 79% of the sample. Among the invisible categories, CHA/EDCA predominated (46.5%), followed by nursing technicians and other technicians (25.3%), support, maintenance, and cleaning workers (22.3%), and management and surveillance workers in health (5.9%). The socioeconomic and work characteristics of "invisible" workers varied according to their occupations (Table 1 – supplementary material).

The sociodemographic profile did not differ significantly between the groups. Notably, the highest percentage of males was found in health management and surveillance positions. A lack of higher education predominated among the groups, except in management and surveillance (61.7% had higher education). Nearly all support, maintenance, and cleaning workers (96.3%) and CHA/EDCA (93.7%) reported incomes of up to two minimum wages.

The effective employment relationship predominated among CHA/EDCA workers (99.2%); while other categories showed high frequencies of temporary contracts. In all groups, more than a third of workers reported performing activities that were not compatible with their positions, with the highest percentage among support, maintenance, and cleaning workers (41.8%). The majority had been carrying out their work activities for more than five years, with CHA/EDCA workers standing out (96.4%). A significant percentage of workers had a second job, particularly among technicians (32.8%) and those in management and surveillance (40.4%). Most workers reported working up to 40 hours per week.

Regarding occupational stressors (Table 1), the highest percentages of high effort were observed among CHA/EDCA workers (43.5%) and technicians (41.8%). Low reward was prevalent in all groups, with a higher incidence among support, maintenance, and cleaning workers (81%). Excessive commitment to work stood out among CHA/EDCA workers (46.3%) and those in management and surveillance (46.8%). The presence of effort-reward imbalance was evident, with a higher percentage observed among CHA/EDCA workers (27.3%).

**Table 1:** Occupational stressors and common mental disorders, by occupation, among "invisible" workers in primary and medium complexity care. Bahia, 2022.

Characteristics		Support, maintenance, and cleaning		CHA/EDCA		Nursing technicians and others		Management and health surveillance		p-value
		n	%	n	%	n	%	n	%	
Effort (770)*										
	Low	124	73.8	203	56.5	114	58.2	33	70.2	0.001
	High	44	26.2	156	43.5	82	41.8	14	29.8	
Reward (743)*										
	High	31	19.0	96	27.8	56	29.8	13	27.7	0.108
	Low	132	81.0	249	72.2	132	70.2	34	72.3	
Excessive work commitment (781)*										
	Absent	132	75.9	195	53.7	141	71.6	25	53.2	<0.001
	Present	42	24.1	168	46.3	56	28.4	22	46.8	
ERI (726)*										
	Balance	127	80.9	245	72.7	142	76.8	35	74.5	0.252
	Imbalance	30	19.1	92	27.3	43	23.2	12	25.5	
Psychological demand (747)*										
	Low	97	58.4	236	67.4	112	59.6	25	58.1	0.122
	High	69	41.6	114	32.6	76	40.4	18	41.9	
Control over the work (727)*										
	High	57	35.0	135	39.7	86	47.0	24	58.5	0.015
	Low	106	65.0	205	60.3	97	53.0	17	41.5	
Social support (750)*										
	High	46	27.7	96	27.4	59	31.2	13	29.5	0.805
	Low	120	72.3	255	72.6	130	68.8	31	70.5	
Demand-control model (694)*										
	Low demand	33	21.3	94	28.7	48	27.7	10	26.3	0.018
	Active work	21	13.5	35	10.7	33	19.1	11	28.9	
	Passive work	58	37.4	127	38.7	54	31.2	13	34.2	
	High demand	43	27.7	72	22.0	38	22.0	4	10.5	
CMD (740)*										
	No	115	70.1	181	52.8	122	64.6	27	61.4	<0.001
	Yes	49	29.9	162	47.2	67	35.4	17	38.6	

* The Ns varied due to information losses for the analyzed variables. CHA/EDCA: Community Health Agents/Endemic Disease Control Agents.

There was a higher prevalence of high psychological demand among management workers (41.9%) and support, management, and cleaning workers (41.6%). The majority reported low control over work, except for management and surveillance workers (58.5% high control). Low social support at work was also prevalent across all strata. Approximately one-third of workers were in a passive work situation (low psychological demand and low control over work).

The prevalence of CMD among "invisible" workers was 39.9%, with a notable occurrence among CHA/EDCA workers (47.2%), followed by management and surveillance (38.6%), technicians (35.4%), and support/maintenance/cleaning staff (29.9%).

In all groups, occupational stressors were associated with CMD at statistically significant levels, except for the reward dimension. The impact of stressors on the association with CMD varied across the investigated groups. Regarding the stressors assessed by ERI, high effort and high commitment, as well as the situation of effort-reward imbalance, were statistically associated with CMD in all groups (Table 2). Considering the dimensions of DCM, high psychological demand was positively associated with CMD only among nursing technicians/other technicians, similar to low control (associated with CMD only in this group). Passive work situation was associated with CMD among support/maintenance and cleaning staff; high demands were associated with CMD for nursing technicians/other technicians and support/maintenance and cleaning staff.

**Table 2:** Association between occupational stressors and common mental disorders, by occupation, among "invisible" workers in primary and medium complexity care. Bahia, 2022.

Characteristics		Common mental disorders											
		Support, maintenance, and cleaning			CHA/EDCA			Nursing technicians and others			Management and health surveillance		
		P	PR	95%CI	P	PR	95%CI	P	PR	95%CI	P	PR	95%CI
Effort													
	Low	23.5	1.00		37.5	1.00		24.8	1.00		26.7	1.00	
	High	43.9	1.87	1.16–3.02	59.2	1.57	1.25–1.98	52.3	2.07	1.39–3.07	64.3	2.41	1.18–4.91
Reward													
	High	32.1	1.00		41.1	1.00		33.3	1.00		33.3	1.00	
	Low	31.2	0.97	0.53–1.76	49.8	1.21	0.92–1.59	36.0	1.08	0.69–1.68	40.6	1.22	0.49–3.01
EWC													
	Absent	21.5	1.00		32.6	1.00		26.7	1.00		26.1	1.00	
	Present	57.5	2.67	1.74–4.12	64.5	1.98	1.56–2.51	60.8	2.27	1.59–3.25	52.4	2.01	0.90–4.47
ERI													
	Balance	25.4			41.7			27.7			28.1		
	Imbalance	48.3	1.89	1.17–3.09	61.4	1.47	1.17–1.85	61.5	2.21	1.53–3.20	66.7	2.37	1.19–4.69
Psychological demand													
	Low	26.4	1.00		49.1	1.00		29.6	1.00		32.0	1.00	
	High	38.2	1.45	0.91–2.29	42.3	0.86	0.66–1.12	45.7	1.54	1.05–2.27	50.0	1.56	0.72–3.32
Control over the work													
	High	23.5	1.00		43.8	1.00		25.9	1.00		36.4	1.00	
	Low	33.7	1.43	0.81–2.52	49.5	1.13	0.88–1.44	44.1	1.70	1.10–2.62	41.2	1.13	0.51–2.50
Social support													
	High	23.8			42.9			34.5			36.4		
	Low	31.9	1.34	0.73–2.45	49.1	1.15	0.87–1.50	37.4	1.08	0.704–1.66	38.7	1.06	0.43–2.61
Demand-control model													
	Low demand	13.8	1.00		44.0	1.00		22.9	1.00		20.0	1.00	
	Active work	40.0	2.90	1.01–8.34	42.4	0.96	0.61–1.53	34.5	1.50	0.70–1.66	50.0	2.50	0.63–9.99
	Passive work	31.5	2.28	0.85–6.15	52.3	1.20	0.90–1.60	35.3	1.54	0.81–2.92	38.5	1.92	0.46–7.94
	High demand	39.5	2.86	1.07–7.65	43.1	0.98	0.68–1.41	54.1	2.36	1.29–4.29	50.0	2.50	0.52–12.14

CHA/EDCA: Community Health Agents/Endemic Disease Control Agents; P: prevalence; PR: prevalence ration; CI: confidence interval; EWC: Excessive work commitment; ERI: Effort-Reward Imbalance.

Considering that the measured occupational stressors overlap in some items of the evaluated dimensions (items of effort and psychological demand), which could lead to overadjustment, separate Poisson Regression analyses were conducted for each of the models (ERI and DCM). The final (adjusted) models revealed that the association between occupational stressors and CMD varied according to occupations:

1. EWC, ERI, and psychological demand were associated with CMD among support/maintenance/cleaning workers;
2. EWC and ERI remained in the final models among CHA/EDCA;
3. EWC, ERI, and low control over work were associated with CMD among technicians;
4. Among management and surveillance workers, only ERI remained associated with CMD (Table 3 and 4).

**Table 3:** Final regression model*, associating occupational stressors (effort-reward imbalance) with common mental disorders, by occupation, among "invisible" workers in primary and medium complexity care. Bahia, 2022.

Characteristics	CMD							
	Support, maintenance, and cleaning		CHA/EDCA		Nursing technicians and others		Management and health surveillance	
	PR	95%CI	PR	95%CI	PR	95%CI	PR	95%CI
Excessive work commitment	2.01	1.02–3.95	2.11	1.45–3.07	1.77	1.05–3.01	-	-
E-R imbalance	2.19	1.14–4.18	1.44	1.04–2.01	1.83	1.06–3.14	5.33	1.25–22.59

* Model adjusted for sociodemographic and labor-related covariates that showed statistically significant differences between strata (gender, race/ethnicity, age, education level, marital status, income, working hours, employment status, years in the profession, and having another job). CMD: common mental disorders; CHA/EDCA: Community Health Agents/Endemic Disease Control Agents; PR: prevalence ratio; CI: confidence interval; E-R: effort-reward.

**Table 4:** Final regression model*, association between occupational stressors (demand-control model) and common mental disorders, by occupation, among "invisible" workers in primary and medium complexity care. Bahia, 2022.

Characteristics	CMD							
	Support, maintenance, and cleaning		CHA/EDCA		Nursing technicians and others		Management and health surveillance	
	PR	95%CI	PR	95%CI	PR	95%CI	PR	95%CI
High psychological demand	2.48	1.18–5.22	-	-	-	-	-	-
Low control	-	-	-	-	2.17	1.26–3.71	-	-

* Adjusted model for sociodemographic and labor covariates that showed statistically significant differences between strata (gender, race/color, age, education, marital status, income, working hours, employment status, years of professional experience, and having another job). CMD: common mental disorders; CHA/EDCA: Community Health Agents/Endemic Disease Control Agents; PR: prevalence ratio; CI: confidence interval.

## DISCUSSION

The socioeconomic and labor characteristics differed among the unseen professional categories, with certain groups experiencing clear disadvantages, particularly those responsible for support, maintenance, and cleaning services, as well as CHA/EDCA.

There was a high exposure to occupational stressors and a high prevalence of CMD, with variations between groups, indicating specific exposure to occupational stressors (according to occupations).

EWC, ERI, and psychological demand were associated with CMD among support/maintenance/cleaning workers. Among CHA/EDCA, EWC and ERI remained associated with CMD, while EWC, ERI, and low control over work were associated with CMD among technicians. Among management and surveillance workers, only ERI remained associated with CMD.

The predominance of women in the health sector is longstanding. Studies indicate that approximately 70% of health professional teams are comprised of women, a trend that has remained unchanged during the pandemic, both in Brazil and worldwide^9,19–21^. Among "invisible" workers, there is a predominance of women (72.5%), of black/brown ethnicity (59%), and aged between 36–50 years (50.3%)^9^, findings consistent with our results.

The sexual division of labor remains evident, with command positions predominantly occupied by men, while women predominate in care activities. Studies on the dynamics that support and reproduce certain conceptions about femininity and masculinity indicate that these conceptions shape the sexual division of labor. The socially constructed and reproduced idea that women are "naturally" suited to domestic care activities reinforces the perception and expectation that women are better prepared to perform care-related tasks in professional settings^22^.

The data showed a high prevalence of CMD in all categories of "invisible" workers, consistent with results found in other national studies with HW, which range between 16 and 46.9%^23–28^. The characteristics of work environment, context, and work management contribute significantly to the mental illness of workers^6^. Factors exacerbated by the pandemic must also be considered: long working hours, disrupted sleeping patterns, low pay, multiple employment relationships, and aspects of the work process^29^.

Among CHA/EDCA workers, the prevalence of CMD was higher than in other categories in this study and in other investigations^30,31^. This finding can be attributed to the various challenges these professionals faced during the pandemic, such as the inclusion of new demands, acquisition of new knowledge, improvement of practices, and use of new tools. Additionally, they had to recognize the demands and particularities of the territory under their responsibility^32^. These challenges were compounded by the fear of contamination, the use of new personal protective equipment (PPE), and limited knowledge about a new disease, which could cause tension and overload, impacting the mental health of these workers.

Our findings indicated that specific occupational stressors were related to a higher prevalence of mental disorders. High levels of effort, low reward, EWC, and ERI among HW have been documented in several studies^23,33–36^. In this study, high levels of occupational stressors were observed: high effort, low reward, EWC, ERI, high psychological demand, low control over work, low social support, and highly demanding working conditions.

The challenges experienced during the pandemic contributed to increased exposure to occupational stressors, as working under excessive pressure, new professional demands, inadequate working conditions, and the constant fear of contagion intensely affected healthcare work. The pandemic required workers to confront the situation and be proactive in resolving daily arising cases. Care workers bore the responsibility of providing care and saving lives; surveillance workers were tasked with protection, monitoring, and the challenging control of transmissibility; and managers had to make decisions on collective measures aimed initially at containing the virus and preventing the deaths of those affected^1^.

Exposure to situations of high psychological demands and low control over work is concerning. High psychological demands at work predispose individuals to illness and are described as the variable within the demand-control model most strongly associated with the occurrence of CMD, particularly in HW^36–38^. Control over work is inversely associated with levels of suffering and dissatisfaction arising from work activities^12^. It is believed that having control over one's own work allows workers the autonomy to organize work demands according to their capabilities and skills. This action enhances the positive aspects of work and mitigates the harmful effects caused by excessive demands, potentially reducing mental illness resulting from work^33^.

EWC was associated with CMD in all strata. People who dedicate themselves excessively to work also expect to receive high rewards. During the pandemic, the real increase in demands may have heightened expectations regarding rewards for the work carried out. Consequently, the lack of expected recognition and appreciation can lead to frustration, dissatisfaction, and psychological illness^33^. Unfair labor exchanges are directly associated with mental illness^23,33,35^, a fact evidenced in this study, where the ERI situation remained associated with CMD among support/maintenance/cleaning staff, CHA/EDCA, and technicians.

Low control was associated with CMD among nursing technicians. Workers who have the opportunity to exercise some level of control over their work can better align the demands of their profession with their skills, thereby reducing the harmful effects of unfavorable conditions in the work environment^33^. Conversely, the inability to make subjective adjustments increases pressure on workers, leading to intense suffering.

High psychological demand remained associated with CMD only among support/maintenance/cleaning staff. It is worth noting that, in the context of the pandemic, demands were exacerbated by the need to adapt work methods, use of PPE, meet increased care demands, and manage heavier workloads^21^. Additionally, there was an increased requirement for cleaning and disinfecting environments and reorganizing service flows, which may have contributed to the heightened demands in this occupational group.

This study generated a set of information that clearly signals the need for attention to mental health in healthcare work: a large proportion of "invisible" workers were affected by CMD during the pandemic. However, some limitations of the study need to be considered: the data are based on self-report, which may introduce memory bias and healthy worker bias, as well as the possibility of reverse causality (the events were analyzed at the same point in time, making it impossible to verify causation in advance).

It is worth noting that the study was conducted approximately one year after the start of the pandemic, by which time vaccines were already available, health services were more structured to manage and confront the disease, and professionals were better prepared to manage positive cases, use PPE, and implement protective measures to prevent the spread of the virus.

In this sense, it is believed that the estimates of CMD and its relationship with occupational stressors likely reflect a milder reality compared to the beginning of the pandemic. However, despite these limitations, this study makes important contributions to public health and health planning by analyzing the morbidity profile of specific groups of HW, which have historically been neglected.

The presence of occupational stressors during the COVID-19 pandemic was highlighted, along with their association with mental illness among HW, irrespective of the professional category. It underscores the importance of considering the work profile and the most prevalent psychosocial risks in each occupation to plan and implement measures that enhance the protective dimensions of work and reduce stressful aspects, with the aim of promoting healthier work environments.

## Supplementary Material


