# Supplementary material for: Dimensions of the COVID-19 pandemic: prevalence of common mental disorders in "invisible" health workers and their association with occupational stressors
Source: Rev Bras Epidemiol. 2024 Jul 29;27:e240039. doi: 10.1590/1980-549720240039 (PMC11290769; doi:10.1590/1980-549720240039)
Supplement: Supplementary file 1 [file 1980-5497-rbepid-27-e240039-Suppl01.pdf]

Tabela 1. Características socioeconômicas e do trabalho, segundo ocupação, entre trabalhadores(as) "invisiveis" da atenção básica e da média complexidade. Bahia, 2022.

Variáveis	Apoio, conservação e limpeza		ACS/ACE		Técnicos de enfermagem e outros		Gestão e vigilância em saúde		p-valor
	n	%	n	%	n	%	n	%	
**Sexo (791)***									
Masculino	46	26,3	63	17,1	18	9,0	15	31,9	< 0,001
Feminino	129	73,7	305	82,9	183	91,0	32	68,1	
**Raça/cor da pele (780)***									
Brancos(as)	15	8,6	9	2,5	12	6,1	6	13,0	< 0,001
Pretos(as)/pardos(as)	160	91,4	354	97,5	184	93,9	40	87,0	
**Escolaridade (788)***									
Com nível superior	37	20,9	129	35,4	42	21,0	29	61,7	< 0,001
Sem nível superior	140	79,1	235	64,6	158	79,0	18	38,3	
**Idade (793)***									
Menos de 40 anos	76	43,2	63	17,0	76	37,8	12	26,1	< 0,001
40 anos ou mais	100	56,8	307	83,0	125	62,2	34	73,9	
**Filhos (788)***									
Não	41	23,3	63	17,1	57	28,9	14	29,8	0,006
Sim	135	76,7	305	82,9	140	71,1	33	70,2	
**Situação Conjugal (794)***									
Sem companheiro	82	46,3	142	38,4	96	48,0	15	31,9	0,041
Com companheiro	95	53,7	228	61,6	104	52,0	32	68,1	
**Renda (493)***									
Até 2 salários mínimos	104	96,3	223	93,7	95	83,3	25	75,8	< 0,001
Mais de 2 salários	4	3,7	15	6,3	19	16,7	8	24,2	
**Vínculo (772)***									
Efetivo	88	51,8	360	99,2	83	42,6	21	47,7	< 0,001

Temporário	82	48,2	3	0,8	112	57,4	23	52,3	
**Atividade compatível com o cargo (778)***									
Sim	99	58,2	243	66,9	120	60,6	30	63,8	0,206
Não	71	41,8	120	33,1	78	39,4	17	36,2	
**Tempo de Trabalho (788)***									
Até 5 anos	78	44,3	13	3,6	62	31,0	23	50,0	< 0,001
Mais de 5 anos	98	55,7	353	96,4	138	69,0	23	50,0	
**Outro trabalho (795)***									
Não	154	87,0	352	95,1	135	67,2	28	59,6	< 0,001
Sim	23	13,0	18	4,9	66	32,8	19	40,4	
**Jornada Semanal (783)***									
Até 40 horas	123	70,7	299	81,7	122	61,9	34	73,9	< 0,001
Mais de 40 horas	51	29,3	67	18,3	75	38,1	12	26,1	

***** Os N variaram em função de perdas de informações para as variáveis analisadas.
