# Supplementary material for: Dimensions of the COVID-19 pandemic: prevalence of common mental disorders in "invisible" health workers and their association with occupational stressors
Source: Rev Bras Epidemiol. 2024 Jul 29;27:e240039. doi: 10.1590/1980-549720240039 (PMC11290769; doi:10.1590/1980-549720240039)
Supplement: Translation

# Translation: Dimensões da pandemia de COVID-19: prevalência de transtornos mentais comuns em trabalhadores e trabalhadoras "invisíbilizados" da saúde e sua associação com estressores ocupacionais

**DOI:** 10.1590/1980-549720240039.2

**Authors:** Manuela Matos Maturino, Camila Carvalho de Sousa, Lusicleide Galindo da Silva Moraes, Danyella Santana Souza, Maria Yaná Guimarães Silva Freitas, Tânia Maria de Araújo

**Affiliations:** I Universidade Estadual de Feira de Santana, Núcleo de Epidemiologia – Feira de Santana (BA), Brasil.; II Secretaria da Saúde do Estado da Bahia, Núcleo Regional de Saúde Leste – Salvador (BA), Brasil.; III Hospital Universitário Professor Edgar Santos – Salvador (BA), Brasil.; IV Universidade Estadual de Feira de Santana, Núcleo de Pesquisa Integrado em Saúde Coletiva, Núcleo de Pesquisa e Extensão em Vigilância à Saúde – Feira de Santana, Bahia, Brasil.

**Keywords:** COVID-19, Trabalhador da saúde, Estresse ocupacional, Saúde mental

## Abstract

**Objetivo:**

Avaliar associação entre estressores ocupacionais e transtornos mentais comuns (TMC) entre trabalhadores e trabalhadoras "invisíbilizados" da saúde, no contexto da pandemia de COVID-19.

**Métodos:**

Estudo transversal com amostra probabilística de 1.014 trabalhadores(as) da saúde de três municípios baianos. Os TMC foram avaliados pelo SRQ-20. A escala Desequilíbrio Esforço-Recompensa (DER) e o Modelo Demanda-Controle avaliaram os estressores ocupacionais. Análise descritiva, bivariada e múltipla avaliaram associação entre as variáveis de interesse.

**Resultados:**

A prevalência global de TMC foi de 39,9%, sendo mais elevada entre os Agentes Comunitários de Saúde/Agente de Combate às Endemias — ACS/ACE (47,2%), seguidos pelo pessoal da gestão e vigilância (38,6%), técnicos (35,4%) e pessoal de apoio/conservação/limpeza (29,9%). A associação entre estressores ocupacionais e TMC variou entre as ocupações: 1. Comprometimento excessivo com o trabalho (CET), DER e demandas psicológicas associaram-se aos TMC entre trabalhadores de apoio/conservação/limpeza; 2. CET e DER estavam associadas aos TMC entre ACS/ACE; 3. CET, DER e baixo controle sobre o trabalho associaram-se aos TMC entre os técnicos; 4. Entre trabalhadores de gestão e vigilância, apenas o DER estava associado aos TMC.

**Conclusões:**

Estressores ocupacionais tiveram papel relevante no adoecimento mental, com variação entre os estratos de ocupação, demandando atenção, acompanhamento e controle.

## INTRODUÇÃO

Em meio a situações de crise sanitária, os trabalhadores(as) da saúde (TS) que atuam na linha de frente tendem a sentir-se desamparados(as), sobrecarregados(as) de atividades, física e psicologicamente afetados. Intensificação do ritmo e demanda do trabalho; mudanças drásticas na rotina de trabalho; ausência ou limitação de suporte e condições laborais desfavoráveis, com elevação do nível de exposição ao risco de adoecer e morrer, foram situações evidenciadas com a emergência de uma nova doença, a COVID-19^1^.

Os serviços de saúde, essenciais ao combate e enfrentamento de uma pandemia, estavam entre os poucos serviços que se mantiveram presenciais durante todo o período. No Brasil, o Sistema Único de Saúde (SUS) é o principal responsável por desenvolver ações e prestar serviços de saúde para mitigar a ocorrência da COVID-19. Numa crise sanitária, como uma pandemia, é da organização e de ações de enfrentamento desempenhadas por esses trabalhadores que depende a vida de populações inteiras^2,3^.

O adoecimento psíquico dos TS tem sido foco de diversos estudos nos últimos anos, com destaque para os transtornos mentais comuns (TMC), os quais caracterizam-se por sintomas como insônia, ansiedade, fadiga, irritabilidade, humor depressivo, dificuldade de concentração e queixas somáticas^4^. Apesar de não configurarem uma categoria diagnóstica específica, possui correspondência com critérios de transtornos presentes na Classificação Internacional de Doença (CID-10) e no Manual Diagnóstico e Estatístico (DSM)^5^.

A elevada demanda psicossocial é uma das características mais frequentemente identificadas como associadas aos efeitos negativos sobre a saúde mental dos TS, com destaque para os estressores ocupacionais^1,6^. A dimensão psicossocial decorre da natureza do trabalho desenvolvido, seja devido ao contato direto com pacientes de difícil manejo, longas jornadas de trabalho ou receio de erros durante o cuidado e precarização das condições de trabalho que são vivenciadas como fontes estressantes do trabalho, gerando sobrecarga e estresse crônico^6,7^. Todos esses fatores foram exacerbados pela pandemia, tornando os TS mais vulneráveis ao adoecimento psíquico.

Apesar da elevada exposição ocupacional das categorias ocupacionais atuantes no setor da saúde e da sua relevância para o funcionamento dos serviços, uma parcela significativa desses trabalhadores é invisibilizada, pouco reconhecida e desconsiderada em estudos que avaliam agravos à saúde dos TS. Esses trabalhadores têm sido chamados de "trabalhadores invisíbilizados", os quais, embora desenvolvam atividades importantes, desempenham funções pouco valorizadas e pouco lembradas na sociedade, muitas vezes "invisíbilizados" diante da própria equipe, suas instituições e da sociedade em geral^8,9^.

Este estudo centra-se na análise desses trabalhadores "invisíbilizados" da saúde, que foram categorizados em quatro grupos, considerando as demandas ocupacionais:

1. Trabalhadores de apoio, conservação e limpeza (condutores de ambulância, manutenção, apoio operacional, equipe da limpeza, cozinha e administração);
2. Técnicos (técnicos e auxiliares de enfermagem, de saúde bucal, de radiologia, de laboratório e análises clínicas);
3. Agentes Comunitários de Saúde (ACS) e Agentes de Combate às Endemias (ACE); e
4. Gestão e vigilância em saúde (sanitaristas, fiscais sanitários, técnicos da vigilância, apoiadores institucionais, gerentes e coordenadores).

Este estudo focaliza a saúde mental dos TS e a relação entre características psicossociais do trabalho e o adoecimento mental observado durante a pandemia. Assim, o estudo objetivou avaliar a associação entre estressores ocupacionais e ocorrência de transtornos mentais comuns entre trabalhadores "invisíbilizados" da saúde, no contexto da pandemia da COVID-19.

## MÉTODOS

Estudo transversal realizado em três municípios do interior da Bahia, vinculado ao projeto de pesquisa "Vigilância e Monitoramento de Doenças Infecciosas no Setor Saúde", desenvolvido com recursos financeiros do CNPq 427045/2016-9, aprovado pelo Comitê de Ética em Pesquisa com Seres Humanos, sob o protocolo CAAE 90204318.2.0000.0053.

Amostra definida em procedimentos de etapas sucessivas: lista nominal dos trabalhadores em atividade; estimativa do tamanho amostral; definição do tamanho amostral; estratificação da amostra; sorteio dos(as) trabalhadores(as) (SPSS, versão 23.0).

Para o cálculo do tamanho amostral, empregou-se o número total de trabalhadores (4.849) nos três municípios, incidência de acidentes de trabalho entre TS de 42%, 95% de confiança e precisão de 3%, resultando em uma amostra mínima necessária de 857 trabalhadores. A esse número, acresceu-se 20%, levando-se em consideração possíveis perdas e recusas, resultando na amostra final de 1.028 trabalhadores.

Este estudo explora a relação entre estressores ocupacionais e transtornos mentais entre os trabalhadores e trabalhadoras "invisíbilizados" da saúde. Não foram incluídos nesta análise: médicos, enfermeiros, odontólogos, fisioterapeutas, psicólogos, nutricionistas, educadores físicos, terapeutas ocupacionais e pedagogos.

A coleta ocorreu entre abril/2021 e abril/2022, por meio de entrevistas face a face nos locais de trabalho, utilizando-se questionário elaborado a partir de revisão de literatura, composto por 8 blocos:

1. Identificação geral;
2. Informações gerais sobre o trabalho;
3. Condições do ambiente/local de trabalho;
4. Características psicossociais do trabalho e saúde mental;
5. Atividades domésticas;
6. Hábitos de vida e aspectos relacionados à saúde;
7. Arboviroses; e
8. Violência.

O controle de qualidade dos dados e a digitação do banco de dados foram realizados por equipe treinada. Medidas de biossegurança foram adotadas para coleta durante a pandemia.

Os TMC, variável desfecho, foram avaliados pelo *Self-ReportingQuestionnaire* (SRQ-20). No rastreio dos TMC, foi adotado ponto de corte de sete ou mais respostas positivas para mulheres e cinco ou mais para homens, como recomendado em estudo de validação do SRQ-20^10^.

Os aspectos psicossociais do trabalho, estressores ocupacionais, foram avaliados por dois modelos: Desequilíbrio Esforço-Recompensa (ERI – *Effort-RewardImbalance*), desenvolvido por Siegrist^11^, e Modelo Demanda-Montrole (MDC), proposto por Karasek^12^ (instrumentos de mensuração testados em população trabalhadora brasileira, apresentando bom desempenho).

O Modelo ERI^11^ avalia esforço (energia despendida para realização do trabalho, envolve gasto físico e psíquico) e recompensa no trabalho (retornos como ganhos financeiros, percepção de reconhecimento e respeito no ambiente laboral, expectativas de promoção, compatibilidade do cargo ocupado com a formação e sentimento de justiça nas relações). O modelo inclui uma terceira dimensão (comprometimento excessivo com o trabalho), que corresponde a um fator intrínseco, subjetivo, que se caracteriza pelo esforço excessivo, visando reconhecimento e aprovação^13^. O ERI contém 23 itens [escala de esforço (effort): 6 itens; recompensa (reward): 11 itens e comprometimento excessivo com o trabalho (over-commitment): 6 itens], respondidos numa escala de concordância que varia de 1 (discordo fortemente) a 4 (condordo fortemente). O escore de cada escala é avaliado pelo somatório dos seus respectivos itens. O segundo tercil foi utilizado como ponto de corte para dicotomização das escalas propostas para composição de esforço (baixo/alto), recompensa (baixa/alta) e comprometimento excessivo (ausente/presente) (Quadro 1). O escore de desequilíbrio esforço-recompensa foi calculado pela fórmula: ERI=Escore de esforço x fator de correção/escore das recompensas. Valores acima de 1 foram considerados como situação de desequilíbrio esforço-recompensa.

O MDC^12^ destaca dois elementos como centrais no trabalho: a demanda psicológica relacionada às exigências psicológicas enfrentadas pelo trabalhador durante a execução de suas atividades laborais (ritmo de trabalho, tempo suficiente para a sua realização, volume excessivo de trabalho, dentre outros) e o controle do trabalhador sobre o próprio trabalho, incluindo autonomia no processo de decisão^14^. A combinação desses elementos identifica quatro situações de trabalho: baixa exigência (alto controle/baixa demanda), trabalho ativo (alta demanda/alto controle), trabalho passivo (baixa demanda/baixo controle) e alta exigência (alta demanda/baixo controle).

A principal hipótese desse modelo é que o trabalho em alta exigência é a situação de maior risco psicossocial à saúde física e mental^13^. O apoio social configura-se como uma terceira dimensão, posteriormente incluída no MDC^15^, acreditando-se que o auxílio na realização das tarefas por parte de colegas e superiores, a integração social e a confiança no grupo atuam como modelador do desgaste à saúde.

O *JobContentQuestionnaire* (JCQ)^16^ é o instrumento desenhado para medir as escalas do MDC. Estudo de validação em trabalhadores formais e informais no Brasil identificou bom desempenho global do instrumento para investigar aspectos psicossociais do trabalho^16^. As escalas do JCQ foram dicotomizadas considerando-se a mediana como ponto de corte para definição de categorias alto(a) e baixo(a) (Quadro 1), estabelecendo-se as situações específicas de trabalho: baixa exigência, trabalho ativo, trabalho passivo e alta exigência^12,14^.

**Quadro 1:** Dimensões do modelo demanda-controle e modelo desequilíbrio esforço-recompensa, número de itens, variação dos escores e pontos de corte utilizados.

Dimensões		N^o^ de itens	Variação	Ponto de corte	
				Mediana	2^o^ tercil
Modelo demanda-controle					
	Demanda psicológica	5	22–68	42,0	
	Controle sobre o trabalho	9	32–92	62,0	
	Apoio social no trabalho	6	6–28	18,0	
Modelo desequilíbrio esforço-recompensa					
	Esforço	6	6–24		15
	Recompensa	11	19–42		30
	Comprometimento excessivo	6	6–24		16

Além dos estressores ocupacionais, mensurados pelo JCQ e ERI, incluiu-se na análise covariáveis socioeconômicas (sexo, idade, escolaridade, raça/cor da pele, situação conjugal, filhos, renda) e laborais (jornada de trabalho, vínculo empregatício, ocupação, tempo de atuação na profissão, atividades compatíveis com o cargo).

Para a caracterização geral da amostra, realizou-se análises descritivas, considerando os grupos de ocupações definidas previamente dos trabalhadores "invisíbilizados". A análise de associação entre estressores ocupacionais e ocorrência de TMC foi estratificada pelas ocupações, sendo calculadas as razões de prevalências (RP), intervalos de confiança (IC) de 95% e os valores de p pelo teste χ^2^ de Pearson (programas *Statistical Program for the Social Sciences* 23.0/SPSS 15.0 e *OpenEpi* 3.0).

Os modelos finais de regressão foram estimados separadamente para os grupos de estressores (desequilíbrio esforço-recompensa e modelo demanda controle) e pelos grupos ocupacionais analisados. A regressão de Poisson com variância robusta foi utilizada para estimativas das razões de prevalência, intervalos de confiança de 95% e valores de p na análise múltipla (multivariável)^17,18^.

Na seleção das variáveis para a análise múltipla foi empregado o teste qui-quadrado de Pearson (X²), considerando-se todas as variáveis de modo não condicional. O nível de significância para entrada no modelo multivariado foi de 25% com utilização do teste de razão de verossimilhança. Para seleção das variáveis, empregou-se o método *backward,* adotando-se o critério de significância de 5% para permanência no modelo final. O diagnóstico de qualidade de ajuste do modelo final foi feito pelo teste de Hosmer e Lemeshow, Curva ROC e padrão de observações influentes. O programa utilizado nesta etapa foi *Data Analysis and Statistical Software* (STATA), versão 12.0.

## RESULTADOS

Amostra constituída por 1.014 TS, dos quais, 795 estavam em ocupações invisibilizadas, representando 79% da amostra. Dentre as categorias invisibilizadas, predominaram ACS/ACE (46,5%), seguidos pelos técnicos de enfermagem e outros técnicos (25,3%), trabalhadores de apoio, conservação e limpeza (22,3%) e trabalhadores da gestão e vigilância em saúde (5,9%). As características socioeconômicas e laborais dos trabalhadores "invisíbilizados" variaram segundo as ocupações (Tabela 1 – material suplementar).

O perfil sociodemográfico não diferiu significativamente entres os grupos. Registra-se que o maior percentual do sexo masculino estava nos cargos de gestão e vigilância em saúde. Ausência de nível superior de escolaridade predominou entre os grupos, exceto gestão e vigilância (61,7% nível superior). Renda de até dois salários mínimos referida pela quase totalidade dos trabalhadores de apoio, conservação e limpeza (96,3%) e ACS/ACE (93,7%).

O vínculo efetivo de trabalho predominou entre os ACS/ACE (99,2%); nas demais categorias, elevadas frequências de contratos temporários. Em todos os grupos, mais de um terço dos trabalhadores referiu desenvolver atividades que não eram compatíveis com o cargo, com maior percentual entre os trabalhadores de apoio, conservação e limpeza (41,8%). A maioria referiu desenvolver as atividades laborais há mais de 5 anos, destacando-se ACS/ACE (96,4%). Houve um percentual expressivo de trabalhadores com duplo vínculo entre técnicos (32,8%) e gestão e vigilância (40,4%). A maioria referiu uma jornada semanal de trabalho de até 40 horas.

Com relação aos estressores ocupacionais (Tabela 1), os percentuais mais elevados de alto esforço estavam entre ACS/ACE (43,5%) e técnicos (41,8%). Baixa recompensa prevaleceu em todos os grupos, sendo mais frequente entre os trabalhadores de apoio, conservação e limpeza (81,%). Comprometimento excessivo com o trabalho destacou-se nos ACS/ACE (46,3%) e gestão e vigilância (46,8%). O desequilíbrio esforço-recompensa foi verificado, com maior percentual nos ACS/ACE (27,3%).

**Tabela 1:** Estressores ocupacionais e transtornos mentais comuns, segundo ocupações, entre trabalhadores(as) "invisíbilizados" da atenção básica e da média complexidade. Bahia, 2022.

Variáveis		Apoio, conservação e limpeza		ACS/ACE		Técnicos de enfermagem e outros		Gestão e vigilância em saúde		p-valor
		n	%	n	%	n	%	n	%	
Esforço (770)*										
	Baixo	124	73,8	203	56,5	114	58,2	33	70,2	0,001
	Alto	44	26,2	156	43,5	82	41,8	14	29,8	
Recompensa (743)*										
	Alta	31	19,0	96	27,8	56	29,8	13	27,7	0,108
	Baixa	132	81,0	249	72,2	132	70,2	34	72,3	
Comprometimento excessivo com o trabalho (781)*										
	Ausente	132	75,9	195	53,7	141	71,6	25	53,2	<0,001
	Presente	42	24,1	168	46,3	56	28,4	22	46,8	
ERI (726)*										
	Equilíbrio	127	80,9	245	72,7	142	76,8	35	74,5	0,252
	Desequilíbrio	30	19,1	92	27,3	43	23,2	12	25,5	
Demanda psicológica (747)*										
	Baixa	97	58,4	236	67,4	112	59,6	25	58,1	0,122
	Alta	69	41,6	114	32,6	76	40,4	18	41,9	
Controle sobre o trabalho (727)*										
	Alto	57	35,0	135	39,7	86	47,0	24	58,5	0,015
	Baixo	106	65,0	205	60,3	97	53,0	17	41,5	
Apoio social (750)*										
	Alto	46	27,7	96	27,4	59	31,2	13	29,5	0,805
	Baixo	120	72,3	255	72,6	130	68,8	31	70,5	
Modelo demanda-controle (694)*										
	Baixa exigência	33	21,3	94	28,7	48	27,7	10	26,3	0,018
	Trabalho ativo	21	13,5	35	10,7	33	19,1	11	28,9	
	Trabalho passivo	58	37,4	127	38,7	54	31,2	13	34,2	
	Alta exigência	43	27,7	72	22,0	38	22,0	4	10,5	
TMC (740)*										
	Não	115	70,1	181	52,8	122	64,6	27	61,4	<0,001
	Sim	49	29,9	162	47,2	67	35,4	17	38,6	

* Os Ns variaram em função de perdas de informações para as variáveis analisadas. ACS/ACE: Agentes Comunitários de Saúde/Agente de Combate às Endemias.

Constatou-se maior prevalência de alta demanda psicológica entre os trabalhadores da gestão (41,9%) e de apoio, conservação e limpeza (41,6%). A maioria relatou baixo controle sobre trabalho, com exceção da gestão e vigilância (58,5% alto controle). O baixo apoio social no trabalho também foi predominante em todos os estratos. Aproximadamente um terço dos trabalhadores estava em situação de trabalho passivo (baixa demanda psicológica e baixo controle sobre o trabalho).

A prevalência de TMC entre os trabalhadores "invisíbilizados" foi de 39,9%, com destaque para ACS/ACE (47,2%), seguidos pela gestão e vigilância (38,6%), técnicos (35,4%) e pessoal de apoio/conservação/limpeza (29,9%).

Em todos os grupos, estressores ocupacionais associaram-se aos TMC, a níveis estatisticamente significante, com exceção da dimensão recompensa. O papel dos estressores na associação com TMC variou nos grupos investigados. Com relação aos estressores avaliados pelo ERI, esforço elevado e alto comprometimento, bem como a situação de desequilíbrio esforço-recompensa, estavam estatisticamente associados aos TMC em todos o grupos (Tabela 2). Considerando as dimensões do MDC, elevada demanda psicológica estava associada positivamente aos TMC apenas entre os técnicos de enfermagem/outros técnicos, situação semelhante a do baixo controle (associado aos TMC apenas neste grupo). Situação de trabalho passivo associou-se aos TMC entre pessoal de apoio/conservação e limpeza; alta exigência estava associada aos TMC para técnicos de enfermagem/outros técnicos e apoio/conservação e limpeza.

**Tabela 2:** Associação entre estressores ocupacionais e transtornos mentais comuns, segundo ocupação, entre trabalhadores(as) "invisíbilizados" da atenção básica e da média complexidade. Bahia, 2022.

Variáveis		Transtornos mentais comuns											
		Apoio, conservação e limpeza			ACS/ACE			Técnicos de enfermagem e outros			Gestão e vigilância em saúde		
		P	RP	IC95%	P	RP	IC95%	P	RP	IC95%	P	RP	IC95%
Esforço													
	Baixo	23,5	1,00		37,5	1,00		24,8	1,00		26,7	1,00	
	Alto	43,9	1,87	1,16–3,02	59,2	1,57	1,25–1,98	52,3	2,07	1,39–3,07	64,3	2,41	1,18–4,91
Recompensa													
	Alta	32,1	1,00		41,1	1,00		33,3	1,00		33,3	1,00	
	Baixa	31,2	0,97	0,53–1,76	49,8	1,21	0,92–1,59	36,0	1,08	0,69–1,68	40,6	1,22	0,49–3,01
CET													
	Ausente	21,5	1,00		32,6	1,00		26,7	1,00		26,1	1,00	
	Presente	57,5	2,67	1,74–4,12	64,5	1,98	1,56–2,51	60,8	2,27	1,59–3,25	52,4	2,01	0,90–4,47
ERI													
	Equilíbrio	25,4			41,7			27,7			28,1		
	Desequilíbrio	48,3	1,89	1,17–3,09	61,4	1,47	1,17–1,85	61,5	2,21	1,53–3,20	66,7	2,37	1,19–4,69
Demanda psicológica													
	Baixa	26,4	1,00		49,1	1,00		29,6	1,00		32,0	1,00	
	Alta	38,2	1,45	0,91–2,29	42,3	0,86	0,66–1,12	45,7	1,54	1,05–2,27	50,0	1,56	0,72–3,32
Controle sobre o trabalho													
	Alto	23,5	1,00		43,8	1,00		25,9	1,00		36,4	1,00	
	Baixo	33,7	1,43	0,81–2,52	49,5	1,13	0,88–1,44	44,1	1,70	1,10–2,62	41,2	1,13	0,51–2,50
Apoio social													
	Alto	23,8			42,9			34,5			36,4		
	Baixo	31,9	1,34	0,73–2,45	49,1	1,15	0,87–1,50	37,4	1,08	0,704–1,66	38,7	1,06	0,43–2,61
Modelo demanda-controle													
	Baixa exigência	13,8	1,00		44,0	1,00		22,9	1,00		20,0	1,00	
	Trabalho ativo	40,0	2,90	1,01–8,34	42,4	0,96	0,61–1,53	34,5	1,50	0,70–1,66	50,0	2,50	0,63–9,99
	Trabalho passivo	31,5	2,28	0,85–6,15	52,3	1,20	0,90–1,60	35,3	1,54	0,81–2,92	38,5	1,92	0,46–7,94
	Alta exigência	39,5	2,86	1,07–7,65	43,1	0,98	0,68–1,41	54,1	2,36	1,29–4,29	50,0	2,50	0,52–12,14

ACS/ACE: Agentes Comunitários de Saúde/Agente de Combate às Endemias; P: prevalência; RP: razão de prevalência; IC: intervalo de confiança; CET: Comprometimento excessivo com o trabalho; ERI: Desequilíbrio Esforço-Recompensa (Effort-Reward Imbalance).

Considerando-se que os estressores ocupacionais mensurados se sobrepõem em alguns itens das dimensões avaliadas (itens de esforço e demanda psicológica), o que poderia ocasionar superajustamento, as análises de Regressão de Poisson foram conduzidas separadamente para cada um dos modelos (ERI e MDC). Os modelos finais obtidos (ajustados) mostraram que a associação entre estressores ocupacionais e TMC variou entre as ocupações:

1. CET, DER e demanda psicológica associaram-se aos TMC entre trabalhadores de apoio/conservação/limpeza;
2. CET e DER permaneceram nos modelos finais entre ACS/ACE;
3. CET, DER e baixo controle sobre o trabalho associaram-se aos TMC entre os técnicos;
4. Entre trabalhadores de gestão e vigilância, apenas o DER permaneceu associado aos TMC (Tabela 3 e 4).

**Tabela 3:** Modelo final de regressão*, de associação entre estressores ocupacionais (desequilíbrio esforço-recompensa) e transtornos mentais comuns, segundo ocupação, entre trabalhadores(as) "invisíbilizados" da atenção básica e da média complexidade. Bahia, 2022.

Variáveis	TMC							
	Apoio, conservação e limpeza		ACS/ACE		Técnicos de enfermagem e outros		Gestão e vigilância em saúde	
	RP	IC95%	RP	IC95%	RP	IC95%	RP	IC95%
Comprometimento excessivo com o trabalho	2,01	1,02–3,95	2,11	1,45–3,07	1,77	1,05–3,01	-	-
Desequilíbrio E-R	2,19	1,14–4,18	1,44	1,04–2,01	1,83	1,06–3,14	5,33	1,25–22,59

* Modelo ajustado pelas covariáveis sociodemográficas e laborais que apresentaram diferenças estatisticamente significantes entre os estratos (sexo, raça/cor, idade, escolaridade, situação conjugal, renda, jornada de trabalho, vínculo empregatício, tempo de atuação na profissão e possuir outro trabalho). TMC: transtornos mentais comuns; ACS/ACE: Agentes Comunitários de Saúde/Agente de Combate às Endemias; RP: razão de prevalência; IC: intervalo de confiança; E-R: esforço-recompensa.

**Tabela 4:** Modelo final de regressão*, de associação entre estressores ocupacionais (modelo demanda-controle) e transtornos mentais comuns, segundo ocupação, entre trabalhadores(as) "invisíbilizados" da atenção básica e da média complexidade. Bahia, 2022.

Variáveis	TMC							
	Apoio, conservação e limpeza		ACS/ACE		Técnicos de enfermagem e outros		Gestão e vigilância em saúde	
	RP	IC95%	RP	IC95%	RP	IC(95%)	RP	IC95%
Alta demanda psicológica	2,48	1,18–5,22	-	-	-	-	-	-
Baixo controle	-	-	-	-	2,17	1,26–3,71	-	-

* Modelo ajustado pelas covariáveis sociodemográficas e laborais que apresentaram diferenças estatisticamente significantes entre os estratos (sexo, raça/cor, idade, escolaridade, situação conjugal, renda, jornada de trabalho, vínculo empregatício, tempo de atuação na profissão e possuir outro trabalho). TMC: transtornos mentais comuns; ACS/ACE: Agentes Comunitários de Saúde/Agente de Combate às Endemias; RP: razão de prevalência; IC: intervalo de confiança.

## DISCUSSÃO

As características socioeconômicas e laborais variaram entre as categorias profissionais invisibilizadas, com desvantagens evidentes para alguns grupos, especialmente para o pessoal responsável pelos serviços de apoio, conservação e limpeza e os ACS/ACE.

Verificou-se elevada exposição aos estressores ocupacionais e alta prevalência de TMC, com variação entre os grupos, com exposições específicas aos estressores ocupacionais (segundo ocupações).

CET, DER e demanda psicológica associaram-se aos TMC entre trabalhadores de apoio/conservação/limpeza. Entre os ACS/ACE, CET e DER permaneceram associados aos TMC, enquanto CET, situação de DER e baixo controle sobre o trabalho associaram-se aos TMC entre os técnicos. Entre trabalhadores de gestão e vigilância, apenas o DER permaneceu associado aos TMC.

O predomínio de mulheres no trabalho do setor saúde é histórico. Estudos descrevem que cerca de 70% das equipes de profissionais de saúde são mulheres, não sofrendo alteração no contexto da pandemia, no Brasil e no mundo^9,19–21^. Em trabalhadores "invisíbilizados", evidencia-se predomínio de mulheres (72,5%), pretos/pardos (59%) e idade entre 36–50 anos (50,3%)^9^, dados concordantes com nossos resultados.

Evidencia-se relações ainda intactas da divisão sexual do trabalho; postos de comando permanecem ocupados, majoritariamente, pelos homens, enquanto as mulheres predominam nas atividade de cuidados. Estudos sobre as dinâmicas que sustentam e reproduzem determinadas concepções acerca do feminino e do masculino apontam que essas concepções edificam a divisão sexual do trabalho. A ideia, socialmente construída e reproduzida, de que as mulheres são "naturalmente" afeitas às atividades relacionadas aos cuidados domésticos consolida percepções e expectativas de que as mulheres estão melhor preparadas para desenvolver as ações de cuidado também nas atividades profissionais^22^.

Os dados evidenciaram alta prevalência de TMC em todas as categorias de trabalhadores "invisíbilizados", o que condiz com os resultados encontrados em outros estudos nacionais com TS, variando entre 16 e 46,9%^23–28^. As características do ambiente laboral, contexto e gestão do trabalho contribuem significativamente para o adoecimento psíquico de trabalhadores^6^. Deve-se considerar, ainda, fatores que foram foram exacerbados no contexto da pandemia: longas jornadas de trabalho, padrão de sono, baixa remuneração, mais de um vínculo empregatício e aspectos do processo de trabalho^29^.

Entre os ACS/ACE, a prevalência de TMC foi superior às demais categorias deste estudo e também de outras investigações^30,31^. Tal achado pode ser explicado pelo fato de a pandemia ter trazido diversos desafios a esses profissionais, como inclusão de novas demandas, aquisição de saberes, aperfeiçoamento de práticas e utilização de novas ferramentas, além do reconhecimento de demandas e particularidades do território de sua responsabilidade^32^, somados ao medo de contaminar-se, utilização de novos equipamentos de proteção individual (EPIs), bem como pouco conhecimento sobre uma nova doença, que podem acarretar tensões e sobrecargas, influenciando na saúde mental desses trabalhadores.

Nossos achados mostraram que estressores ocupacionais específicos estavam relacionados à maior prevalência de transtornos mentais. Níveis elevados de alto esforço, baixa recompensa, CET e DER entre TS têm sido evidenciados em diversos estudos^23,33–36^. Neste estudo, observa-se elevados níveis dos estressores ocupacionais: alto esforço, baixa recompensa, CET, DER, alta demanda psicológica, baixo controle sobre o trabalho, baixo apoio social e condições de trabalho em alta exigência.

Os desafios vivenciados na pandemia contribuíram para elevar a exposição aos estressores ocupacionais, uma vez que trabalhar sob excessiva pressão, novas exigências profissionais, condições de trabalho inadequadas e medo permanente de contágio afetaram intensamente o trabalho em saúde. A pandemia impôs aos trabalhadores a necessidade de enfrentar a situação e ser proativo na resolutividade dos casos que surgiam diariamente, cabendo ao trabalhador da assistência o cuidado e o dever de salvar vidas; à vigilância, a responsabilidade da proteção, monitoramento e o desafiador controle da transmissibilidade; e ao gestor, a tomada de decisões de medidas coletivas, que inicialmente visavam conter o vírus e evitar a morte dos afetados^1^.

A exposição a situações de altas demandas psicológicas e baixo controle sobre o trabalho é preocupante, já que a alta demanda psicológica no trabalho predispõe ao adoecimento e tem sido descrita como a variável do modelo demanda-controle que mais fortemente se associa à ocorrência de TMC, especialmente em TS^36–38^. O controle sobre o trabalho associa-se inversamente aos níveis de sofrimento e insatisfação decorrentes da atividade laboral^12^. Acredita-se que o controle sobre o próprio trabalho possibilita ao trabalhador autonomia para organizar as exigências do trabalho de acordo com as suas capacidades e habilidades; esta ação amplia aspectos positivos do trabalho e diminui os efeitos nocivos causados pelas demandas excessivas, podendo reduzir o adoecimento mental decorrente do trabalho^33^.

O CET estava associado aos TMC em todos os estratos. Pessoas que se dedicam excessivamente ao trabalho, alimentam também a expectativa de que receberão elevadas recompensas. Na situação de pandemia, o aumento real das demandas pode ter elevado também as expectativas com relação às recompensas pelo trabalho desenvolvido. Assim, a ausência do reconhecimento esperado — da valorização que não chega — pode causar frustração, insatisfação e, consequentemente, adoecimento psíquico^33^. Trocas laborais injustas estão diretamente associadas ao adoecimento mental^23,33,35^. Fato evidenciado neste estudo, onde situação de DER permaneceu associada aos TMC entre o pessoal de apoio/conservação/limpeza, os ACS/ACE e os técnicos.

O baixo controle esteve associado aos TMC entre os técnicos de enfermagem. O trabalhador que tem a possibilidade de desenvolver algum nível de controle sobre o seu trabalho amplia as possibilidades de modelar as exigências da profissão às suas habilidades, reduzindo os efeitos nocivos das condições desfavoráveis vivenciadas no ambiente de trabalho^33^. Do contrário, a impossibilidade de arranjos subjetivos amplia a pressão sobre as pessoas, produzindo intenso sofrimento.

A alta demanda psicológica permaneceu associada aos TMC apenas entre o pessoal de apoio/conservação/limpeza. Cabe ressaltar que, no contexto da pandemia, as exigências foram exacerbadas, com necessidade de adequações das formas de trabalho, utilização de EPIs, ampliação da demanda de atendimentos, ampliação de carga horária^21^. Além disso, houve a exigência aumentada de limpeza e desinfecção dos ambientes e reorganização dos fluxos de atendimento, fatores que podem ter contribuído para elevar as demandas nesse grupo ocupacional.

Este estudo gerou um conjunto de informações que, claramente, sinalizam a necessidade de atenção à saúde mental no trabalho em saúde: boa parte dos trabalhadores "invisíbilizados" estava afetada pelos TMC, no contexto da pandemia. Porém, algumas limitações do estudo precisam ser consideradas: os dados são baseados em autorrelato, podendo haver viés de memória, viés do trabalhador sadio, além da possibilidade de causalidade reversa (os eventos foram analisados num mesmo ponto do tempo, impossibilitando verificação de antecedência).

Cabe destacar que o estudo foi realizado aproximadamente um ano após o início da pandemia, quando já havia a oferta de vacinas, os serviços de saúde estavam mais estruturados para o manejo e enfrentamento da doença e os profissinais estavam mais preparados para manejar os casos positivos, utilizar os EPIs e implementar medidas de proteção para evitar a propagação do vírus.

Neste sentido, acredita-se que as estimativas de TMC e sua relação com os estressores ocupacionais provavelmente reflitam uma realidade mais amena quando comparada com o início da pandemia. No entanto, apesar dessas limitações, este estudo apresenta contribuições importantes para a saúde pública e o planejamento em saúde, ao analisar o perfil de morbidade de grupos específicos de TS, historicamente negligenciados.

A presença, durante a pandemia da COVID-19, dos estressores ocupacionais foi evidenciada, bem como sua associação com o adoecimento mental entre os TS, independentemente da categoria profissional. Ressalta-se a necessidade de considerar o perfil laboral e os riscos psicossociais mais prevalentes em cada ocupação, a fim de planejar e implementar medidas que elevem as dimensões protetoras do trabalho e que reduzam os aspectos estressores, visando promover ambientes laborais mais saudáveis.
